# Biodegradation of plastic waste by yellow mealworms (*Tenebrio molitor* larvae)

**DOI:** 10.7717/peerj.20429

**Published:** 2026-01-08

**Authors:** Wissarut Srisakvarangkool, Panjamaphon Chanthasena, A’liyatur Rosyidah, Phongsakorn Ganta, Supavadee Kerdtoob, Nawarat Nantapong

**Affiliations:** 1 School of Preclinical Sciences, Institute of Science, Suranaree University of Technology, Nakhon Ratchasima, Thailand; 2 Faculty of Allied Health Sciences, Nakhonratchasima College, Nakhon Ratchasima, Thailand; 3 Research Center for Vaccine and Drugs, National Research and Innovation Agency (BRIN), Bogor, West Java, Indonesia

**Keywords:** Enterobacter xiangfangensis, Polyvinyl chloride, Low-density polyethylene, Polypropylene, Polyethylene terephthalate

## Abstract

**Background:**

Plastics are very widely used worldwide, and most of these are not degradable, resulting in global environmental concerns. Plastic usage is growing faster than it did in the past, especially during the COVID-19 outbreak. Global plastic waste associated solely with this pandemic was estimated to be 8.4 ± 1.4 million tons in 2021, exacerbating the existing global burden of plastic, estimated at 9 billion tons produced up to 2017. Some insects can break down plastic polymers, and their intestinal microorganisms play an important role in the process. The purpose of this study was to investigate the biodegradation of several types of plastics by yellow mealworms (*Tenebrio molitor* larvae) and identify the intestinal bacteria engaged in the process.

**Methods:**

In this study, a total of 140 g of mealworms (±1,050 individuals) were divided into seven groups consisting of approximately 150 larvae, and assigned to different plastic feeding conditions, *i.e.* polyvinyl chloride (PVC), polyethylene (PE), polystyrene (PS), polypropylene (PP), and polyethylene terephthalate (PET) for 30 days. The consumption rate of plastic, mealworm total live biomass retention, and the life cycle of mealworms were observed. The gut microorganisms of mealworms with the highest rate of plastic consumption were isolated and identified using 16S rRNA gene sequencing. Their potential for plastic degradation was assessed by testing their capability to grow in a minimal medium with PVC film serving as the sole carbon source.

**Results:**

After a month, PVC was consumed by mealworms more than other plastic sources, as evidenced by their regular life cycle and total live biomass retention (94.8702 ± 2.4278%). A bacterial strain (MG06) with potential PVC-degrading capability was isolated from the guts of the mealworms and was identified as *Enterobacter xiangfangensis* based on 16S rRNA gene sequencing and phylogenetic analysis. The strain demonstrated PVC-dependent growth and survival, indicating its potential to utilize PVC as a carbon source. To the best of our knowledge, no information regarding *E. xiangfangensis* concerning plastic degradation has been disclosed. This work reports the first evidence suggesting that this bacterium species may contribute to the biodegradation of PVC.

## Introduction

Plastics are man-made synthetic polymers that are extensively used globally due to their manufacturing simplicity, accessibility, and broad applicability (Bahl et al., 2021). The immense use of plastic and the lack of degradability led to waste accumulation and environmental concerns (Nayanathara, Pilapitiya & Ratnayake, 2024). Overconsumption of plastics during the COVID-19 pandemic was unavoidable in preventing contamination, ranging from plastic-based personal protective equipment (PPE) to plastic packaging for individual wrap items. The monthly use of plastic-based PPE, including disposable facial masks and medical gloves, has been reported to be approximately 129 billion and 65 billion pieces, respectively (Prata et al., 2020). This surge in single-use plastic extended to packaging, driven by consumer demand and behavioral changes such as panic buying, stockpiling, and a reliance on takeaway services and online shopping (Parashar & Hait, 2021). At the peak of the pandemic in June 2020, daily plastic waste generation reached approximately 1.6 million tons (Ganguly & Chakraborty, 2024). It was further extrapolated that a total of 8.4 ± 1.4 million tons of pandemic-associated plastic waste were generated in 2021 (Mukherjee et al., 2025; Peng et al., 2021). This new wave of waste compounded a pre-existing global plastic crisis, where cumulative production had already reached 9 billion tons by 2017 (Nikiema & Asiedu, 2022).

Currently, the ecosystem and biodiversity are under threat due to plastic pollution, as reflected in numerous documentaries (Adam, 2019; Deia, 2019) and several scientific literatures (Ayassamy, 2025; Lakhiar et al., 2024; Yang et al., 2024). Plastic affects wildlife in three major ways: (1) plastic ropes, nets, and bags cause entanglement that affects at least 344 species, leaving them unable to feed and survive, eventually leading to their extinction; (2) direct consumption of plastics fragments or or indirect consumptionthrough the feeding of prey that contains plastic; and (3) physical interactions such as collisions, obstructions, or abrasions (Cedervall et al., 2012; Oliveira et al., 2013). Thus, there is an urgent need to address the global plastic issues. Nowadays, a variety of recycling methods are employed, such as mechanical recycling, which involves reintroduction plastic waste into the new fabrication plastic, or chemical recycling, which involves procedures linked to turning plastic waste into valuable chemicals. However, these methods have a number of drawbacks, including the production of harmful byproducts, production lower-value plastics quality, and high energy requirements for some processes (Bulak et al., 2021; Khairul Anuar et al., 2025).

Biodegradation of plastic is an emerging field in microbiology that breaks down plastic waste using single enzymes or microbial consortia (Akram et al., 2024). In the case of plastic degradation, there are two type of plastics: non-biodegradable and degradable plastics. Non-biodegradable plastics are fossil-based synthetic polymers that are extremely resistant to enzymes due to their long chain of hydrocarbons with high stability of charges along their length (Reddy, 2008). There are several types of non-biodegradable plastics according to their monomer’s chemical composition *e.g.*, polyvinyl chloride (PVC), polypropylene (PP), polystyrene (PS), low/high-density polyethylene (LDPE/HDPE), and polyethylene terephthalate (PET), due to their durability and resistance to various conditions (Krueger, Harms & Schlosser, 2015; Parashar & Hait, 2021). On contrary, biodegradable plastics are plastics that are either bio-based or fossil-based and can be degraded by microorganisms (Ahmed et al., 2018). Biodegradable plastics such as polybutylene adipate terephthalate (PBAT), poly (lactic acid) (PLA), polyhydroxyalkanoates (PHAs), starch blends, polybutylene succinate (PBS), polycaprolactone (PCL) and regenerated cellulose, have been extensively developed within the past decades (Kelaniyagama, Gannoruwa & Nilmini, 2024; Sathiaseelan et al., 2024). However, biodegradable plastics remain problematic despite their potential. Although legislative efforts have been established to standardized definitions and specifications of biodegradable plastics, the complicated descriptions and coverage of bioplastics and biodegradable plastics (*e.g.*, bio-based, biodegradable, compostable, oxo-biodegradable plastic, *etc*.) are confusing to the public. The word “bioplastic” is often mistakenly used as a synonym for “biodegradable plastic”. This is incorrect, as these terms are not interchangeable. A bioplastic is defined as a plastic that is biobased, biodegradable, or both. It is crucial to note that “biobased” (derived from biological resources) and “biodegradable” (susceptible to microbial decomposition) are independent properties. For example, bio-polyethylene (Bio-PE) is biobased but not biodegradable, PCL is biodegradable but not biobased, and PHA are both biobased and biodegradable (Dilkes-Hoffman et al., 2019). Therefore, misleading labels can lead to increased usage, improper disposal and scattered waste (Choe et al., 2021; Goel et al., 2021). While laboratory studies confirm the complete mineralization of biodegradable plastics under controlled settings, non-ideal environmental conditions often lead to incomplete degradation and microplastic formation (Tao et al., 2024; Zhu & Wang, 2020). Moreover, bioplastic production also requires significant resources and costs (Kim et al., 2023).

Previously, darkling beetle has been used as a generic term to describe beetles in the Tenebrionidae family, and moths in the family Pyralidae were reported to be able to penetrate PE, PVC, and PP films (Newton, 1988). In 2014, Chinese researchers reported isolating a PE-degrading bacterial strain from *Plodia interpunctella* larvae (Indian meal moth), indicating that the larvae may be able to decompose LDPE (Yang et al., 2014). Apart from that, there have been extensive studies focusing on the ability to consume plastics of various insects, especially members from the order Lepidoptera (*e.g.*, *Galleria mellonella* and *Plodia interpunctella*) and Coleoptera (*Tenebrio molitor*, *Tenebrio obscurus, Zhophobas atratus*, *etc*.) (Pivato et al., 2022). Yellow mealworms (*T. molitor* L.) are the larval stage of yellow mealworm beetles, a species of darkling beetles. These ubiquitous insects will pass through complete metamorphosis in approximately 4–5 months (Engel & Moran, 2013; Morales-Ramos et al., 2010). Nowadays, *Tenebrio* sp. larvae, particularly *T. molitor*, are generally used to perform plastic biodegrading research. It has been shown that *T. molitor* larvae are able to consume PS from styrofoam as their sole carbon source (Yang et al., 2015a). Strong evidence suggests the importance of gut microorganisms in the styrofoam-digesting ability of mealworms (Yang et al., 2015b). Furthermore, other types of plastic, like PS, PE, PVC, nylon, PET, PU, and LDPE have also been shown to be degraded and utilized by mealworms’ gut microorganisms (Yang & Wu, 2020; Yang et al., 2023).

To the best of our knowledge, three *Tenebrio* species have been reported to biodegrade plastics, including *Tenebrio opacus,* exclusively native to France (Calmont & Soldati, 2008), and *T. molitor* and *T. obscurus,* which are commercially available worldwide (Ong et al., 2018; Peng et al., 2019). In this study, the mealworm, *T. molitor* larvae, were fed with different types of plastic diets, including PVC, LDPE, PP, and PET, to evaluate their effects on live biomass retention and identify the gut microbes possibly involved in the biodegradation process.

## Materials & Methods

### Materials

The tested plastics were obtained from daily consumable items. Polypropylene from yogurt containers (PP-C), polypropylene from clear plastic bags (PP-B), PET from water plastic bottles, LDPE from opaque plastic bags, and PVC from plastic wrap. Late-instar larvae mealworms (*T. molitor* L.) and rice bran for the control group were purchased from a commercial store, Nakhon Ratchasima, Thailand. A single batch of mealworms was employed in this study to control for potential cohort-related variations and to maintain experimental consistency.

Liquid carbon-free basal medium (LCFBM) containing 0.7 g/L of KH_2_PO_4_, 0.7 g/L of K_2_HPO_4_, 0.7 g/L of MgSO_4_ ⋅ 7H_2_O, 1.0 g/L of NH_4_NO_3_, 0.005/L g of NaCl, 0.002 g/L of FeSO_4_ ⋅ 7H_2_O, 0.002 g/L of ZnSO_4_ ⋅ 7H_2_O, and 0.001 g/L of MnSO_4_ ⋅ H_2_O with a final pH of 7.0 was used to isolate and cultivate microbial strain. The nutrient broth (NB) medium was prepared by dissolving 3 g/L of beef extract, 10 g/L of bacteriological tryptone, and 5 g/L of NaCl in deionized water with a final pH of 7.0. Saline water (SW) was prepared by dissolving 8.5 g/L of NaCl (0.85%, w/v) in deionized water, and a final pH was adjusted to 7.2. All media were sterilized by autoclaving at 121 °C for 15 min.

### Mealworm rearing and plastic feeding test

The mealworms were divided into seven groups (20 g, ±150 mealworms per group). Five groups of mealworms were fed with PVC (1.1650 ± 0.000$\overline{0}$8 g), PP-B (1.1667 ± 0.0003 g), LDPE (1.1626 ± 0.0001 g), PP-C (1.1670 ± 0.000$\overline{0}$9 g), and PET (1.1650 g), respectively. Meanwhile, group 6 (positive control) was fed with rice bran. Group 7 was used as a negative control without any feed. Groups of mealworms were reared in separated PP plastic containers (12 cm × 8 cm × 3 cm). The plastic pieces were thoroughly cleaned with water and dish soap and then air-dried at room temperature beforehand. Eight drops of water were randomly added to the mealworm containers every two days to provide necessary hydration. All groups were incubated at room temperature for 30 days under a photoperiod of 1 h of light and 23 h of darkness.

### Plastic mass loss measurement

All plastic pieces were drawn out every day to measure their weight. The process started with cleaning plastic thoroughly with a paintbrush to remove molted skin debris. Then, the plastic was weighed using a four-digit weighing balance (Shimadzu model: ATX224). The procedures were carried out when mealworms were exposed to light (1 h of photoperiod), and each plastic was drawn out for approximately 10 min to minimize time without food.

The plastic weight loss is calculated based on the following equation (Bulak et al., 2021):

\begin{eqnarray*}\text{Weight Loss}= \frac{{m}_{i}-{m}_{f}}{{m}_{i}} \times 100\% \end{eqnarray*}

where:

*m*_*i*_ - the initial mass of plastic in grams.

*m*_*f*_ - the final mass of plastic in grams.

### Evaluation of pupation, eclosion rate, and live biomass retention

Mealworm pupae were counted daily for 30 days across all plastic treatment groups, including the positive control (rice bran) and negative control (non-fed) groups. Each pupa was monitored until it emerged as an adult darkling beetle, and any deaths during this transition were recorded.

To evaluate live biomass retention, the total weight of all living larvae, pupae, and adults was measured on day 30. Dead larvae were removed immediately upon discovery and excluded from the final biomass calculation. Although live biomass reduction does not directly reflect survival rates, it can indirectly indicate population dynamics, such as mortality caused by environmental stressors or nutritional deficiencies. The live biomass retention is calculated based on the following equation (Pradhan & Satapathy, 2014; Thayaparan et al., 2013):

\begin{eqnarray*}\text{Live Biomass Retention} \left( \% \right) = \frac{{w}_{f}}{{w}_{i}} \times 100\% \end{eqnarray*}

where:

*w*_*i*_ - the initial total weight of mealworms in each group in grams.

*w*_*f*_ - the final total weight of mealworms, pupae, and adult beetles from each group in grams.

### Isolation of gut microorganisms

Mealworms were surface-sterilized by immersion in 75% ethanol for 1 min and washed twice with sterile normal saline. The guts of mealworms were then extracted. The intestine samples were subsequently placed in a 15 mL centrifuge tube containing five mL saline. After agitation with a vortex mixer at full speed for 5 min, the intestinal tissue was carefully removed with a pipette. The suspension was transferred to a 250 mL Erlenmeyer flask containing 3.5 × 3.5 cm sheet of plastic film and 80 mL of LCFBM (Machona, Chidzwondo & Mangoyi, 2022; Yang et al., 2015b). The flask was incubated on a rotary shaker (120 rpm) at room temperature for 60 days. Afterwards, the residual plastic sheets were removed, and the cultured supernatant was spread on nutrient agar plates and incubated at room temperature for 24 h. The appeared colonies were picked and purified on NA medium. To further verify bacterial growth on PVC as the sole carbon source, an isolated strain was cultured on a fresh LCFBM medium containing a new PVC plastic sheet, minimizing the potential carryover of trace carbon from the gut suspension. The culture was incubated under the same conditions for 60 days.

### 16S rRNA gene sequencing and analysis

The genomic DNA of the isolated gut bacterial was extracted using the freeze-thaw method. Approximately two loops of bacterial colony were transferred to a microcentrifuge tube containing one mL sterile water. Then, the samples were incubated at −80 °C for 5–10 min or until the samples frozen and subsequently thawed at room temperature. The freezing and thawing were repeated 5 times, where each time, samples were completely thawed and mixed thoroughly using vortex mixer. The extracted genome quality was verified by agarose gel electrophoresis. The genome was used as a template for 16S rDNA amplification by polymerase chain reaction (PCR) using universal primers 27F (5′-AGAGTTTGATCMTGGCTCAG-3′) and 1525R (5′-AAGGAGGTGATCCAGCC-3′). The size and purity of PCR products were examined by gel electrophoresis. The band corresponding to 16S rDNA was purified using FavorPrep GEL/PCR Purification Kit (FAVORGEN Biotech Corporation, Ping Tung, Taiwan). The purified fragment was submitted for 16S rRNA sequencing at Macrogen Incorporated, South Korea. The sequences were then aligned with organisms in the EzBioCloud database using pairwise sequence similarity values between a query sequence and the reference sequences employing USEARCH program as an identification engine.

### Statistical analysis

The quantitative and analytical values of the experiments were denoted as mean ± SD. The plastic weight loss measurement and live biomass retention experiment were conducted in triplicate. The normality of the data was tested using the Shapiro–Wilk test. One-way ANOVA was performed with Holm-Sidak’s multiple comparisons *post hoc* test. The linear relationship between plastic consumption and live biomass retention was assessed by computing the Pearson correlation coefficient. A *p* < 0.05 was considered statistically significant.

## Results

### Plastic consumption by mealworms

**Figure 1:**
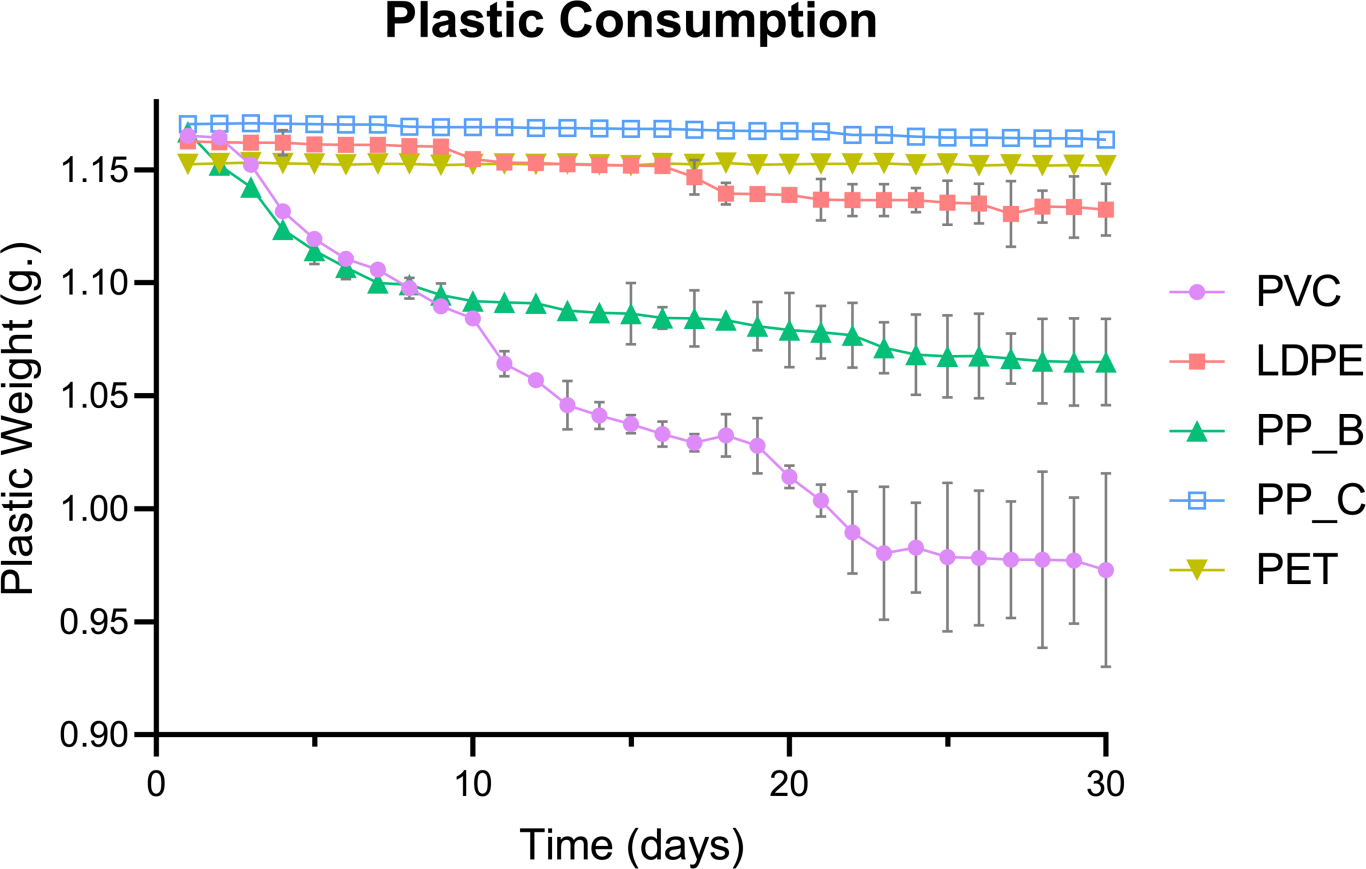
Daily consumption trends of five plastic types by *Tenebrio molitor* larvae over 30 days. Daily plastic consumption rates of five plastic types by *T. molitor* larvae over a 30-day period. Plastic types include polyvinyl chloride (PVC), low-density polyethylene (LDPE), polypropylene plastic bag (PP_B), polypropylene plastic container (PP_C), and polyethylene terephthalate (PET). Data represents the average weight (g) of each plastic type by day, indicating the consumption rate and preference of *Tenebrio* species for different plastics over time. Error bars represent standard deviation across (*n* = 3) replicates.

The consumption of five plastic types by mealworms was quantified over a 30-day period, with untreated plastics to replicate environmental waste conditions. The daily mass changes of five plastic types consumed by mealworms revealed distinct consumption patterns (Fig. 1). PVC-fed larvae exhibited the highest plastic consumption, followed by PP-B and LDPE. In contrast, no apparent mass changes were observed for PP-C or PET, suggesting minimal utilization of these plastics as a food source.

**Figure 2:**
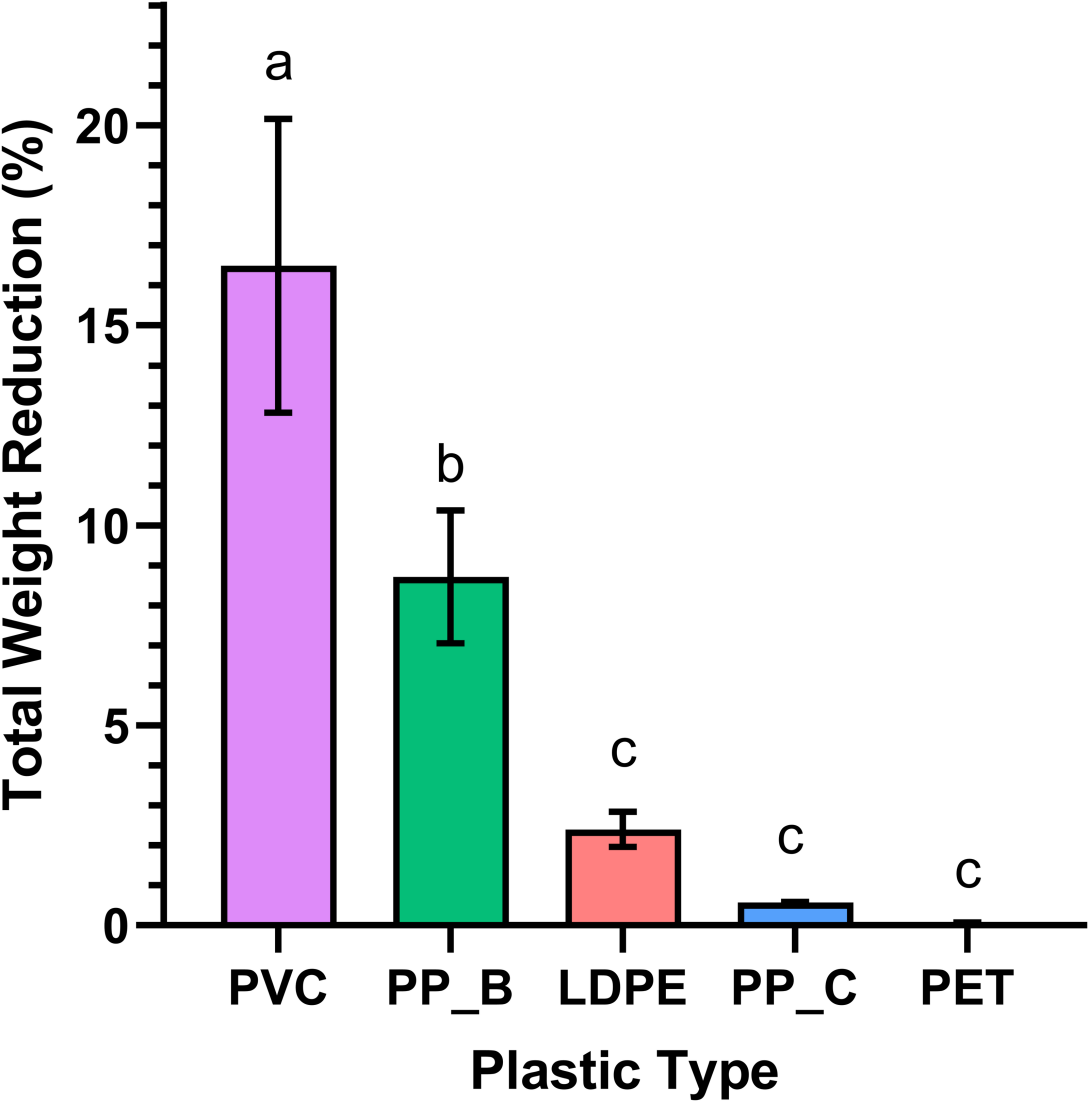
Weight reduction of various plastics by *Tenebrio molitor* larvae over 30 days. Bar chart illustrates the total weight reduction of several plastic types (PVC, LDPE, PP-B, PP-C, and PET) ingested by *T. molitor* larvae. A statistically significant difference in mean weight loss was observed between at least two groups (*p* < 0.0001). Holm-Sidak’s multiple comparison test revealed significant differences between the PVC and PP-B groups (*p* = 0.0019) and between the PP-B and LDPE groups (*p* = 0.0065). Statistically significant differences are denoted by different letters above each group.

Total weight reduction of the plastics, calculated as the mass difference between day 1 and day 30, varied significantly among plastic types (*p* < 0.0001; Fig. 2). PVC showed the greatest weight loss, consistent with its high daily consumption in Fig. 1. *Post hoc* analysis revealed significantly higher consumption of PP-B compared to LDPE (*p* = 0.0065). However, no significant differences were detected between LDPE, PP-C, and PET groups (*p* > 0.05), demonstrating negligible weight reduction throughout the experimental period. This pattern suggests mealworms have limited capacity to consume these polymer types. Together, these results suggest that mealworms preferentially consume PVC, followed by PP-B, while showing limited capacity to degrade LDPE, PP-C, or PET. The significant reduction in PVC and PP-B mass supports their potential as food sources for mealworms.

### Life cycle analysis and live biomass retention

**Figure 3:**
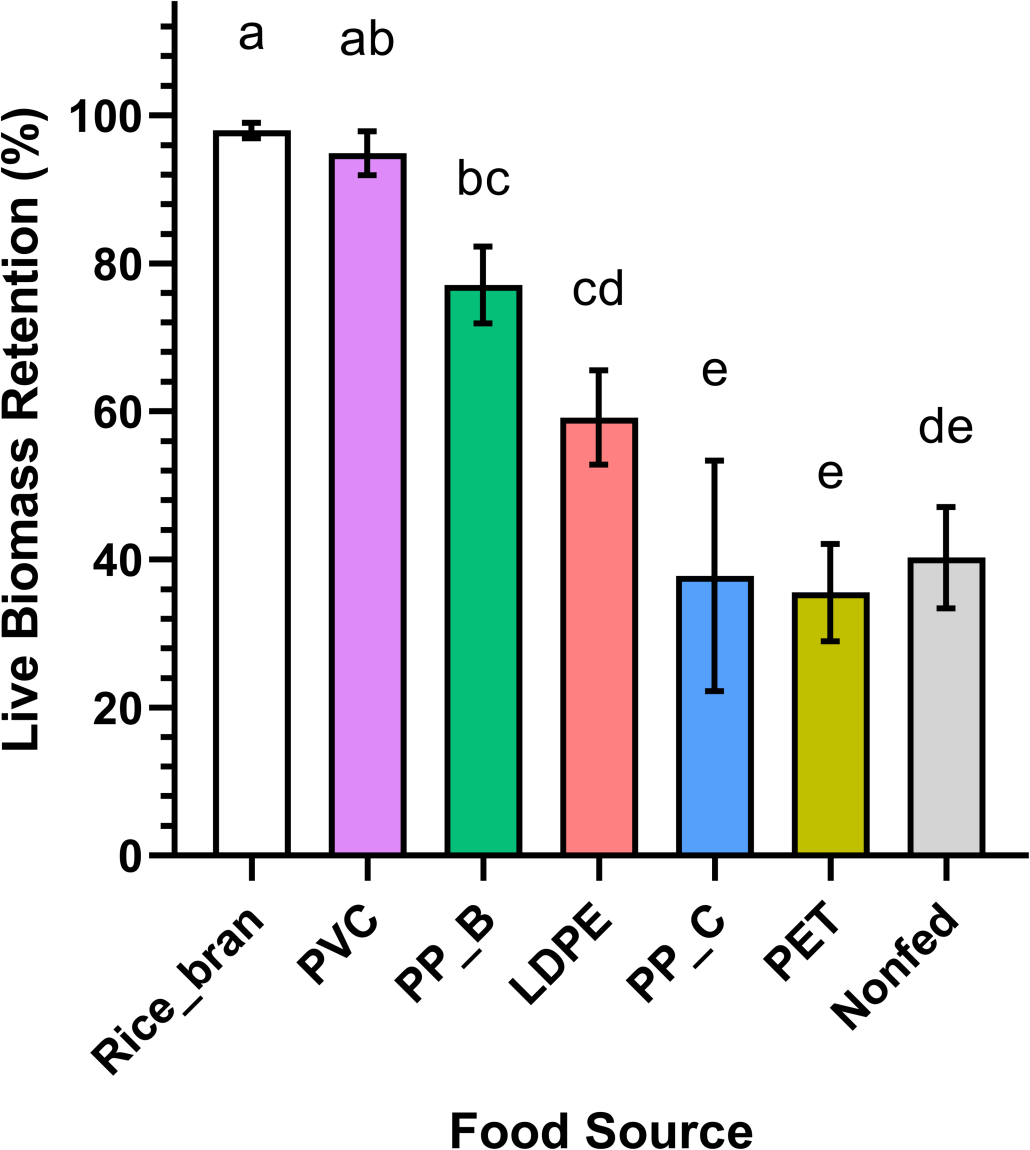
Live biomass retention of *Tenebrio molitor* larvae fed on different plastic types and controls. *T. molitor* larvae live biomass retention across different feeding groups, including rice bran (positive control), PVC, PP-B, PP-C, PET, and a nonfed negative control group. Significant differences in live biomass retention between at least two groups were found by statistical analysis using a one-way ANOVA (*p* < 0.0001). The rice bran and PVC-fed groups showed the highest live biomass retention with no statistically significant difference observed between them (Holm-Sidak’s multiple comparison test, *p* = 0.9483). The PP-C and PET-fed groups, along with the nonfed negative control group, exhibited the lowest live biomass retention, with no significant differences between them (*p* > 0.05). Different lowercase letters above the boxes indicate statistically significant differences between groups.

For each dietary group, the live biomass retention of mealworm was calculated as the percentage change in total weight of larvae, pupae, and adult beetles at day 30 relative to day 1 (Table 1; Fig. 3). The rice bran (positive control) and PVC-fed groups similarly exhibited the highest live biomass retention (97.97% ± 0.86 and 94.87% ± 2.43, respectively) (*p* = 0.9483). This data suggests that PVC is a suitable food source for mealworms, comparable to their natural diet. In contrast, mealworms fed PP-B and LDPE showed intermediate retention (77.07% ± 4.23 and 59.14% ± 5.20), while PP-C, PET, and non-fed groups had the lowest retention (37.79% ± 12.71, 35.53% ± 5.39, and 40.25% ± 5.58, respectively). Notably, PP-C and PET-fed mealworms retained slightly less biomass than the starved control, though differences were not statistically significant (*p* > 0.05). These results imply that mealworms cannot derive sufficient nutrition from PP-C or PET, whereas PVC and PP-B are more readily consumed.

**Table 1:** Live biomass retention of *Tenebrio molitor* larvae fed with different food source. Live biomass retention of *T. molitor* in each group, calculated with the combined weights of mealworms, pupae, and adult darkling beetles. Providing an overview of the viability of *T. molitor* when exposed to various plastic types.

**Food source**	**Initial weight (g)**	**Final weight (g)**	**Biomass retention (%)**
Rice Bran	20.0592 ± 0.0275	19.6522 ± 0.1954	97.9710 ± 0.0281^a^
PVC	20.0593 ± 0.0251	19.0308 ± 0.5068	94.8727 ± 0.0264^ab^
PP-B	20.0585 ± 0.0174	15.4595 ± 0.8572	77.0724 ± 0.0226^bc^
LDPE	20.0532 ± 0.0350	11.8660 ± 1.0638	59.1725 ± 0.0591^cd^
PP-C	20.0599 ± 0.0364	7.5856 ± 2.5662	37.8147 ± 0.0963^e^
PET	20.0305 ± 0.0191	7.1183 ± 1.0847	35.5375 ± 0.0537^e^
Non-Fed	20.0668 ± 0.0139	8.0767 ± 1.1240	40.2492 ± 0.0346^de^

**Figure 4:**
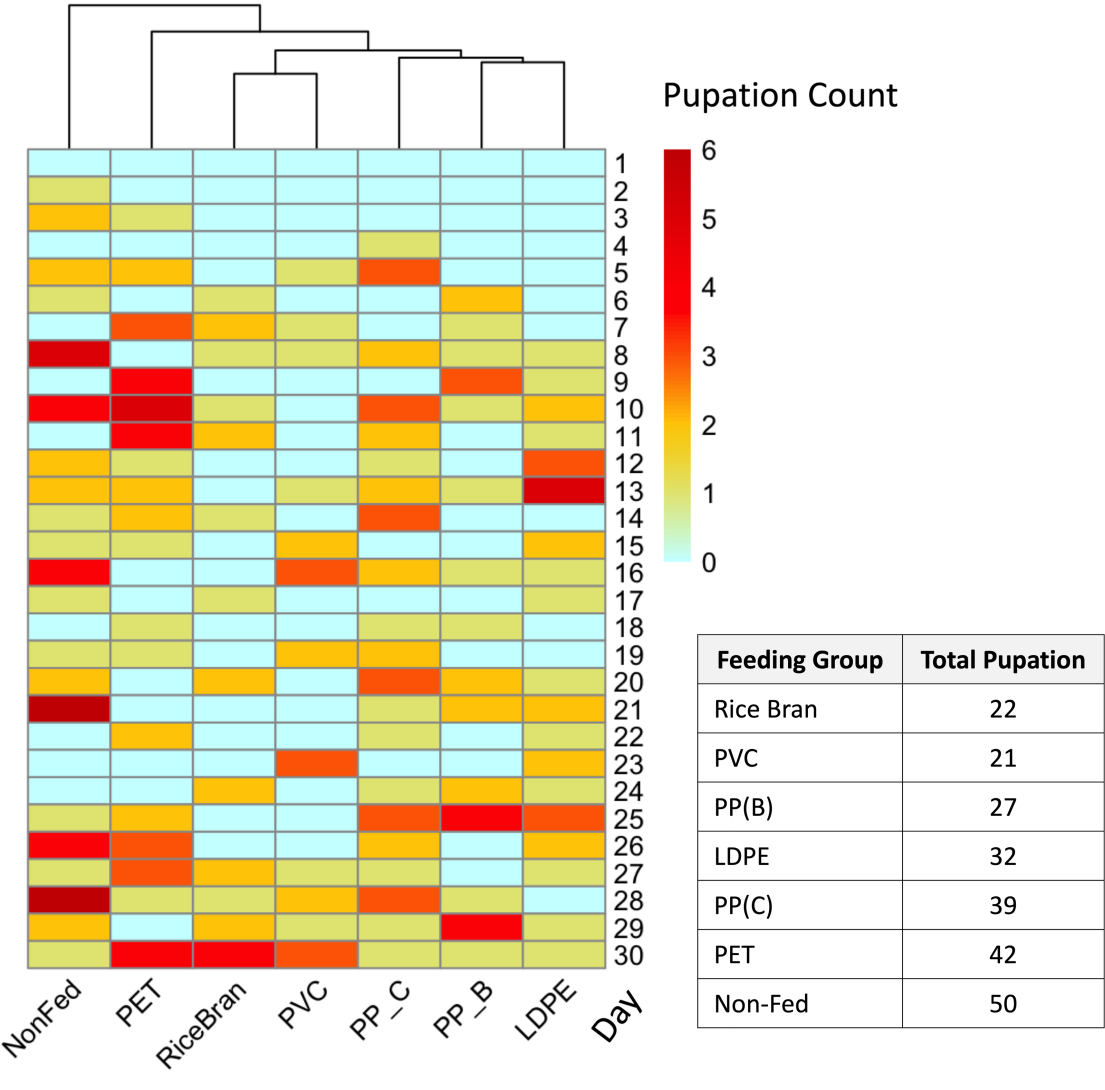
Pupation rate of *Tenebrio molitor* fed with different types of plastic over 30 days. Heatmap representing the pupation rates of *T. molitor* larvae fed on different plastic types over a 30-day period, with darker red indicating higher pupation rates (dark red = 6) and light blue indicating no pupation (0). The dendrogram above the heatmap shows the similarity in daily pupation counts between groups with different food sources, clustering groups with similar pupation responses. The pupation rates of the positive control and PVC-fed groups closely correspond, showing lower rates compared to the PET-fed, PP-C-fed, and nonfed negative control groups. The table on the right shows total pupation counts from each plastic type.

To complement biomass data and further evaluate the dietary effects of different plastics, pupation events were recorded daily across all groups. The pupation results (Fig. 4) showed the rice bran and PVC-fed groups had the lowest total pupation counts (22 and 21 pupae, respectively). In comparison, pupation increased progressively in PP-B (27 pupae), LDPE (32 pupae), PP-C (39 pupae), and PET-fed groups (42 pupae), while the maximum pupation was observed in non-fed controls (50 pupae). This pattern reveals that mealworms fed PVC and rice bran not only showed higher plastic consumption but also developed fewer pupae than those fed PET or PP-C. This inverse relationship between plastic intake and pupal counts aligns with the known biological phenomenon where malnourished mealworms undergo accelerated pupation (Connat et al., 1991; Mini & Prabhu, 1986; Ross, Endersby & Hoffmann, 2016).

### Isolation of gut microorganisms

**Figure 5:**
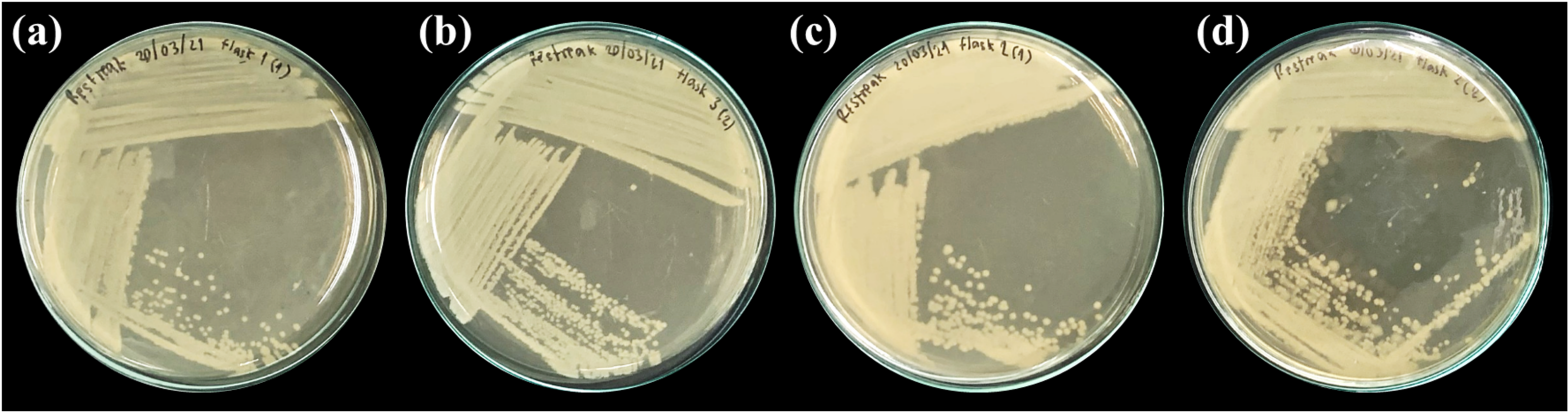
Representative bacterial isolates from the guts of PVC-fed *Tenebrio molitor* larvae. Four selected bacterial isolates grown on nutrient agar, isolated from the gut of *T. molitor* mealworms enriched with PVC diet. The isolates displayed are (A) MG01, (B) MG04, (C) MG06, and (D) MG07.

**Figure 6:**
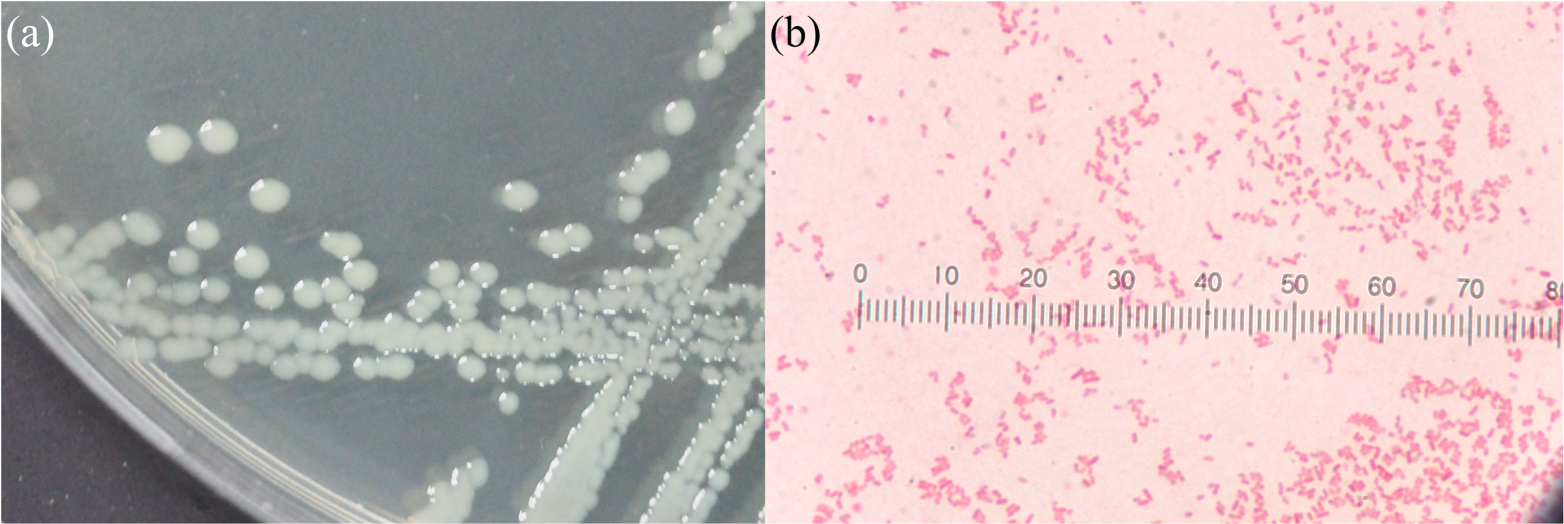
Morphological characteristics of bacterial strain isolated from PVC-fed *Tenebrio molitor* larvae guts. Morphological characteristics of bacterial strain from the gut of PVC-fed *T. molitor*. (A) Colony morphology on nutrient agar plates, with all isolates exhibiting a similar appearance: yellowish-white, opaque, circular, undulated, convex, and smooth. (B) Gram-stained microscopy images of the strain, similarly, showing rod-shaped, Gram-negative bacteria.

**Figure 7:**
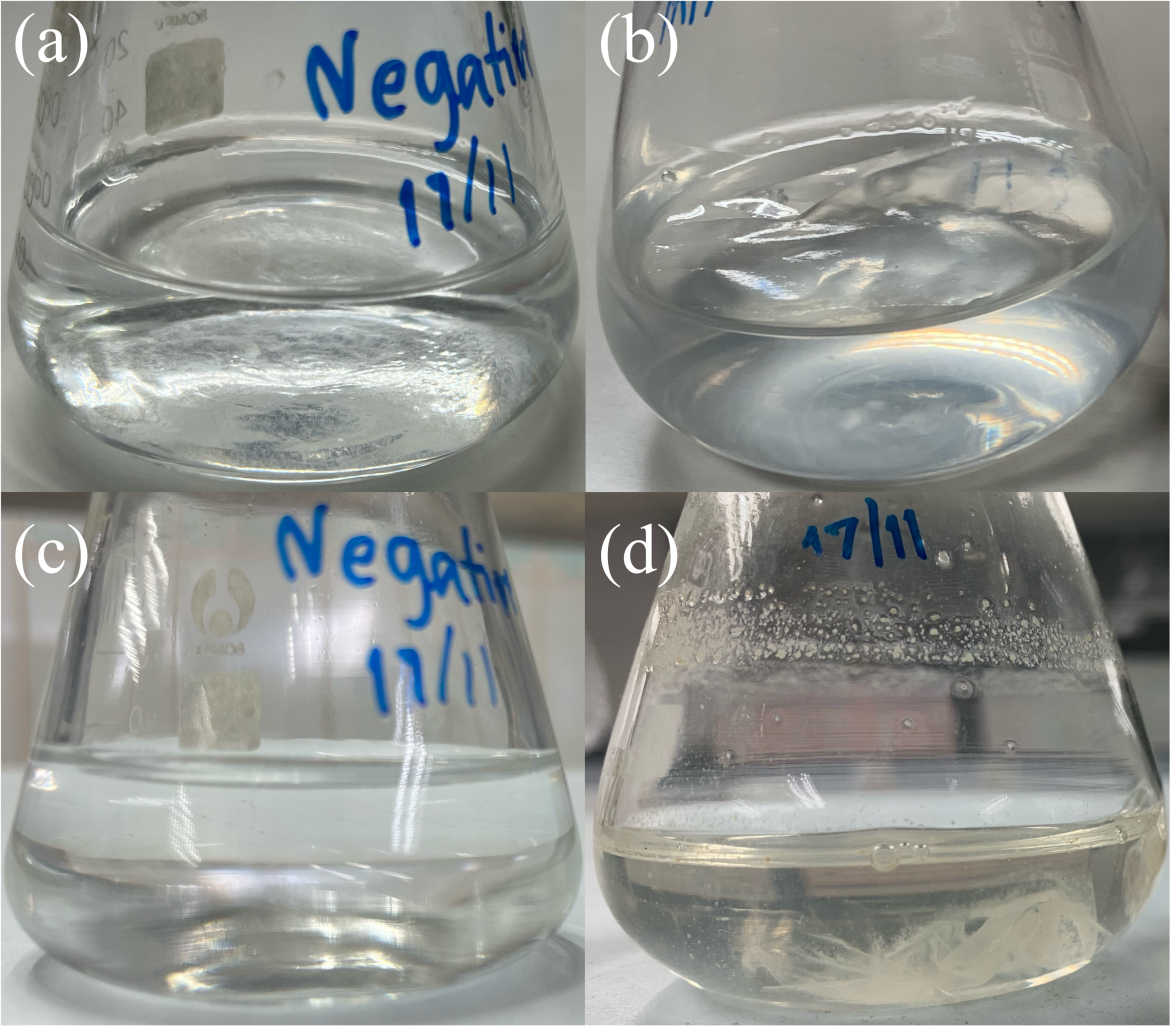
Bacterial attachment on PVC film after 60 days of incubation. Visual comparison of bacterial growth in liquid carbon-free basal medium (LCFBM) without (A, C) and with PVC film (B, D). (A, B) Cultures after 3 days of incubation; (C, D) cultures after 60 days. In the presence of PVC film (B, D), the bacteria appeared to attach to the plastic surface, especially after prolonged incubation (D). No visible attachment or growth was observed in the negative control without PVC (A, C).

The food source that yielded the highest plastic consumption rates by mealworms was used for isolating plastic-utilizing associate-gut microorganisms. Therefore, gut-associated microorganisms capable of utilizing plastic were isolated from PVC-fed mealworms. The gut microbiome extracted from PVC-fed mealworms was enriched with an LCFBM containing PVC film as the sole carbon source. Following enrichment, several bacterial isolates (*e.g.*, MG01, MG04, MG06, and MG07; Fig. 5) were obtained. On nutrient agar plates, all isolates exhibited visually similar colony morphologies, appearing as yellowish-white, opaque, circular, undulated, convex, and smooth (Fig. 6A). Microscopic analysis confirmed that these colonies consisted of rod-shaped, Gram-negative cells (Fig. 6B). All isolates demonstrated the ability to grow in a minimal medium with PVC as the sole carbon source. To simplify further analysis, isolate MG06 was selected as a representative strain. MG06 maintained viability in the PVC-containing medium for 60 days. As shown in Fig. 7, visible growth developed in the PVC-containing flask, while no growth was observed from the PVC-free negative control. Furthermore, dense biofilm formation was observed on the PVC surfaces (Fig. 7D), indicating active bacterial colonization and potential surface modification. To conclusively confirm cell viability and metabolic activity after the 60-day incubation, cultures of strain MG06 from the PVC-containing flasks were subcultured onto nutrient agar. The robust growth of MG06 colonies (Fig. S1) provides direct evidence that living, functional cells were successfully recovered. Most importantly, no growth was observed when the same subculturing process was applied to the negative control (minimal medium with no carbon source). This result confirms that bacterial survival was entirely dependent on the presence of PVC, demonstrating its role as a sole carbon and energy source.

### Molecular identification and phylogenetic analysis of gut microorganisms

**Figure 8:**
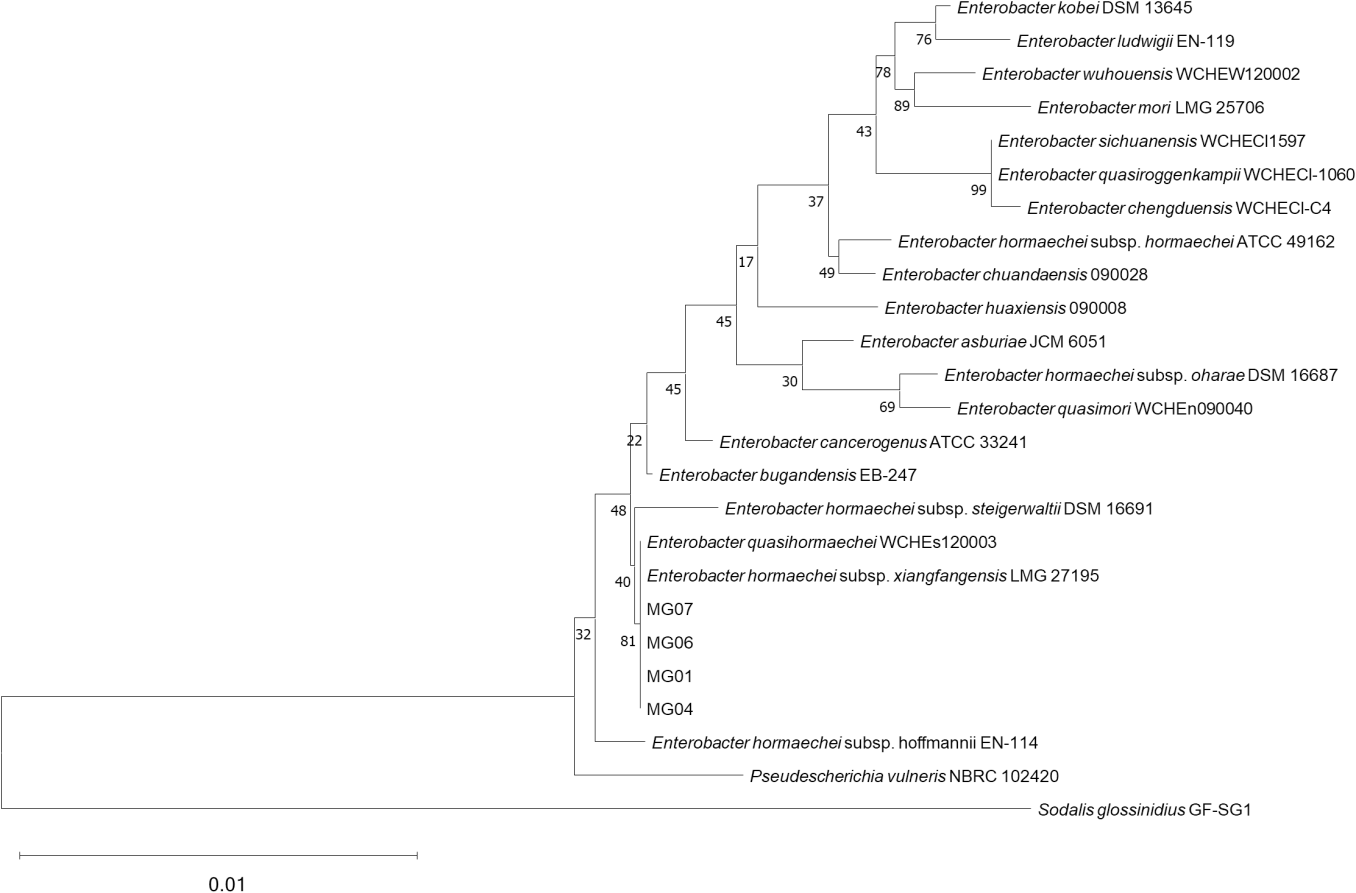
Phylogenetic tree of 16S rRNA gene sequences showing relationships of bacterial isolates from PVC-fed *Tenebrio molitor* larvae guts. Phylogenetic tree constructed using the neighbor-joining algorithm based on 16S rRNA gene sequences, illustrating the relationship between isolated bacteria (MG01, MG04, MG06, MG07) and their related species. *Sodalis glossinidius* was included as an outgroup to root the tree. Bootstrap values on the nodes represent support from 10,000 replicates.

16S rRNA gene analysis showed that all bacterial isolates (MG01, MG04, MG06, and MG07) shared ≥ 99.9% nucleotide similarity with *Enterobacter xiangfangensis* and were placed within the *E. xiangfangensis* clade with high bootstrap support in the neighbor-joining phylogenetic tree (Fig. 8). The high degree of sequence identity (99.93–100%) indicates that these isolates represent a single strain.

## Discussion

Plastics are considered persistent pollutants owing to their resistance to degradation. The accumulation of post-consumer plastic items poses a significant environmental threat (Bozek, Hanus-Lorenz & Rybak, 2017). While the widespread integration of plastic products has undoubtedly improved everyday life, their improper disposal and rapid accumulation have become a pressing global issue (An et al., 2023). Conventional waste management methods like landfilling, incineration, and recycling face challenges due to escalating plastic demand, prompting interest in biodegradation as a sustainable alternative (Jiang et al., 2021). The mealworm *T. molitor* has demonstrated the potential to consume various plastics, including PE, PS, PP, and PVC, as well as vulcanized butadiene-styrene elastomer rubber (Bozek, Hanus-Lorenz & Rybak, 2017). This process is likely mediated by the gut microbiota, digestive enzymes, and salivary enzymes of the mealworm. Similarly, PE degradation has also been observed in *G. mellonella* (Sanluis-Verdes et al., 2022).

Here, we focused on assessing the biodegradation of single-use plastics by *T. molitor*, with a parallel investigation of gut-derived microorganisms. Our study demonstrates that *T. molitor* exhibits distinct consumption preferences for different plastic types, with PVC from plastic wrap showing the highest consumption rates, followed by softer PP-B. While PP-C and PET, which are known to have a hard texture, are used less frequently. This consumption pattern is likely driven by texture-mediated feeding behavior as softer materials more closely resemble natural food sources. Our results align with Jin et al. (2023), who reported that food texture significantly impacts mealworm ingestion rates. Our findings have practical relevance for waste management, suggesting that thinner, softer plastics that common in packaging, are more susceptible to insect-mediated degradation. While previous studies have documented *T. molitor*’s ability to degrade plastics like PE (Brandon et al., 2018), PS (Brandon et al., 2021), polyester polyurethane (Liu et al., 2022), PET (He et al., 2023), and PP (Yang et al., 2021), our work provides new insights into texture-driven consumption differences and potential PVC-degrading gut microbiota. Although some plastics (*e.g.*, PS and PE) are completely biodegraded and mineralized by mealworms (Brandon et al., 2018; Yang et al., 2018), partially degraded PVC still supports normal development, highlighting its viability as a substrate for mealworm-driven plastic breakdown.

In this study, live biomass retention was used as an indicator of population health. The observed weight loss was primarily due to deceased larvae, which were removed upon detection. While live biomass retention does not directly measure survival, it provides insight into population dynamics under different dietary treatments. The effects of plastic consumption on biomass retention across plastic-fed groups were assessed through daily monitoring of mealworm mass changes. The results showed that PVC-fed mealworms maintained the highest live biomass retention among plastic-fed groups, indifferent from rice bran control levels. A significant portion of the live biomass loss likely resulted from natural mortality due to resource competition among larvae, rather than from natural pupation or adult emergence. The rigid nature of PET and PP-C contributes to lower consumption rates by mealworms. In part, this may be due to the tough physical properties of plastic that hindered chewing and ingestion, leading to live biomass retention patterns that resembled those of unfed larvae. Our observation is consistent with previous findings that hard plastic textures (in their study, PE and PVC) are not conducive to feeding and degradation by yellow mealworm (Jin et al., 2023). Future studies should incorporate direct survival tracking in conjunction with live biomass measurements to provide a more comprehensive assessment.

Depending on the types of plastic consumed, the life cycle progression of mealworms was noticeably affected. PVC-fed mealworms showed reduced pupation corresponding to their high consumption, while PET- and PP-C-fed groups exhibited accelerated pupation similar to starved controls, consistent with the well-documented starvation response in late-stage larvae (Mini & Prabhu, 1986; Ross, Endersby & Hoffmann, 2016; Shafiei, Moczek & Nijhout, 2001). The accelerated pupation in PET and PP-C groups confirms these plastics are poor nutritional sources, while the normal development in PVC-fed larvae suggests this material may be more suitable for mealworm-based biodegradation. The pupation count observed in both positive control and PVC-fed groups was similar and lower than in groups fed with PET, PP-C, and the negative control groups. These differential responses between plastic types could guide targeted waste solutions, particularly since PVC’s support of extended larval stages enables greater plastic consumption. Importantly, all pupated larvae across treatment groups successfully eclosed into visually healthy adult beetles, which may potentially reproduce and generate more larvae for the sustainable degradation of PVC plastic. This potential synergy between PVC biodegradation and mealworm farming suggests dual applications in waste management and sustainable insect production, as PVC appears to support near-normal development without life cycle disruption under the conditions tested.

The gut microbiome of mealworms plays an important role in their digestion process. (Jiang et al., 2021). Peng et al. (2020) suggesting that PVC biodegradation specifically depends on gut microbes (Peng et al., 2020). Supporting this, *Klebsiella* sp. strain EMBL-1 was previously isolated from *Spodoptera frugiperda* larvae fed PVC film which revealed proteins like catalase-peroxidase and dehalogenases potentially involved in PVC breakdown (Zhang et al., 2022). In our study, 16S rRNA gene and phylogenetic tree analysis identified intestinal microorganisms from *T*. *molitor*, with isolates MG01, MG04, MG06, and MG07 clustering with a Gram-negative, facultative anaerobic bacterium *E. xiangfangensis*. This finding is notable because *E. xiangfangensis*, first isolated from traditional sourdough (Gu et al., 2014), is capable of utilizing diverse carbon sources (*e.g.*, glycerol, arbutin, cellobiose, lactose, melibiose, sucrose, trehalose, raffinose, gentiobiose, d-lyxose and d-fucose) and tolerating extreme conditions (9% NaCl, 45 °C). Most significantly, our strain survived for 60 days with PVC as their sole carbon source, demonstrating the potential for plastic biodegradation through substrate colonization, a critical first step in microbial degradation (Restrepo-Flórez, Bassi & Thompson, 2014; Tokiwa et al., 2009; Yoshida et al., 2016). These findings underscore the preliminary phase of microbial plastic degradation, where physical association between the microorganism and PVC precedes enzymatic breakdown.

Regarding *Enterobacter*’s plastic degradation capabilities, prior studies have documented *Enterobacter* sp. D1 degrading PE (Ren et al., 2019) and *Enterobacter asburiae* YT1 breaking down PP (Yang et al., 2021). Our work now reveals new functional potential for *E. xiangfangensis* in PVC biodegradation. Although this species was previously found in *Dendroctonus valens* beetles (Cao et al., 2018), no studies have reported its ability to degrade plastics. Our observations of PVC-dependent mealworm growth and bacterial surface attachment suggest a synergistic relationship where mealworms physically fragment PVC, and gut microbes such as *E. xiangfangensis* may enzymatically degrade it. Therefore, mealworms and their gut microorganisms collectively exhibit the possibility of reducing plastic waste in the environment through a complementary partnership where mealworms provide mechanical fragmentation of PVC through chewing, while *E. xiangfangensis* likely facilitates biochemical degradation.

While our approach shares methodological similarities with Machona, Chidzwondo & Mangoyi (2022), our study reveals several significant new insights that advance the field. The work by Machona, Chidzwondo & Mangoyi (2022) focused on examining PE degradation, while our results demonstrate that PVC triggers different biological responses, most notably supporting near-normal developmental progression in mealworms despite being a persistent plastic. The normal development seen in PVC-fed mealworms contrasts with the developmental delays typically reported in PE-fed mealworms (Yang et al., 2021), implying polymer-specific effects. To our knowledge, this study is one of the first to investigate mealworm consumption of single-use plastic waste (*e.g.*, plastic wraps, bags, and food containers), offering data supporting the potential use of mealworm-based solutions to household plastic waste management.

In summary, we report the presence of *E. xiangfangensis* in the gut microbiome of *T. molitor*’s as a novel candidate for PVC biodegradation. Since the PVC biodegradation capabilities of *E. xiangfangensis* have not yet been reported, the current findings not only broaden the microbial diversity involved in plastic degradation but also suggest that polymer-specific microbial communities may be required for the effective breakdown of different plastic types. Specifically, our results demonstrated PVC-dependent microbial growth through two key observations: (1) visible turbidity development and successful recovery of metabolically active cells from the culture media PVC as sole carbon source and (2) PVC surface colonization. However, to conclusively demonstrate PVC degradation, future studies should include (1) GPC analysis to detect molecular weight changes, (2) FTIR spectroscopy to identify specific bond cleavage patterns, (3) respirometry to quantify mineralization through CO_2_ evolution, (4) SEM-EDS to characterize surface modifications at the micron scale, and (5) whole genome analysis study for identifying genes involved in plastic degradation by analyzing the genetic material of microorganisms known to break down PVC plastics. Collectively, this work could advance fundamental knowledge toward developing sustainable, microbiome-assisted solutions for reducing plastic waste.

## Conclusions

The present study invested mealworms (*T. molitor* larvae) fed with various plastics to assess potential plastic consumption and biodegradation by mealworms. This study assessed the effects of plastic ingestion on the mealworm life cycle, followed by isolation and identification of gut microorganisms from plastic-consuming mealworms. The plastics with the highest consumption rates were PVC sourced from plastic wrap, PP-B from a clear plastic bag and LDPE from an opaque plastic bag, respectively. The crucial factor regarding both consumption capability and life cycle normality was plastic texture. Mealworms were capable of breaking down thin and soft-textured polymers reflected by the most plastic mass loss. In this study, the gut-associated bacterium, *E. xiangfangensis*, was isolated from PVC-fed mealworms. However, further in-depth studies are necessary to decipher how *E. xiangfangensis* strains utilize PVC as its carbon source. Since the biodegradation of man-made plastics is currently deemed slow and ineffective, strategically combining the ability of mealworms and their gut microorganisms could potentially facilitate plastic breakdown, reducing critical environmental plastic waste burden.

## Supplemental Information

10.7717/peerj.20429/supp-1 Supplemental Information 1 Viability of *Enterobacter xiangfangensis* MG06 after long-term incubation with PVC as a sole carbon source A robust growth of MG06 colonies on nutrient agar following subculture from a liquid carbon-free basal medium flasks containing PVC after 60 days of incubation, supporting its potential to utilize PVC as a carbon source. No colony formation was observed in the negative control without PVC (not shown), confirming that survival was dependent on the presence of PVC.

10.7717/peerj.20429/supp-2 Supplemental Information 2 Plastic weight raw data Daily measurements of plastic weight (*n* = 3). Plastics were retrieved from feeding setups and weighed to track degradation trends over the 30-day experimental period.

10.7717/peerj.20429/supp-3 Supplemental Information 3 Pupation rate raw data Daily records of pupae counts, with pupae isolated and counted for each experimental group to monitor pupation process.

10.7717/peerj.20429/supp-4 Supplemental Information 4 Live biomass retention results Calculations of live biomass retention based on the initial and final combined weights of mealworms, pupae, and adult darkling beetles (*n* = 3), providing insights into the effects of various plastic diets on live biomass retention.

10.7717/peerj.20429/supp-5 Supplemental Information 5 R code for heatmap construction R script used to generate the heatmap illustrating pupation counts per day.

## Additional Information and Declarations

### DNA Deposition

The following information was supplied regarding the deposition of DNA sequences: The 16S rRNA gene sequences of bacteria isolated from PVC-fed mealworm gastrointestinal tract are accessible via the National Center for Biotechnology Information (NCBI) Genbank database under the accession number PQ637235 to PQ637238. The deposited gene sequences are available at https://www.ncbi.nlm.nih.gov/nuccore/?term=PQ637235:PQ637238[accn].

### Data Availability

The following information was supplied regarding data availability: The supplementary files include the raw data and code used in this study. The plastic weight data consist of daily measurements over 30 days (*n* = 3) for each experimental group, used to analyze degradation trends. The pupation rate data contain daily counts of isolated pupae, which were used for statistical analyses and heatmap construction. Live biomass retention data includes the initial and final weights of mealworms, pupae, and adult beetles (*n* = 3), providing insights into the effects of plastic diets on live biomass retention. Additionally, the R script for heatmap construction from daily pupation counts is provided to allow replication of the clustering and visualization analysis.

## References

[ref-1] Adam S (2019). The plastic problem.

[ref-2] Ahmed T, Shahid M, Azeem F, Rasul I, Shah AA, Noman M, Hameed A, Manzoor N, Manzoor I, Muhammad S (2018). Biodegradation of plastics: current scenario and future prospects for environmental safety. Environmental Science and Pollution Research.

[ref-3] Akram MA, Savitha R, Kinsella GK, Nolan K, Ryan BJ, Henehan GT (2024). Microbial and enzymatic biodegradation of plastic waste for a circular economy. Applied Sciences.

[ref-4] An R, Liu C, Wang J, Jia P (2023). Recent advances in degradation of polymer plastics by insects inhabiting microorganisms. Polymers.

[ref-5] Ayassamy P (2025). Ocean plastic pollution: a human and biodiversity loop. Environmental Geochemistry and Health.

[ref-6] Bahl S, Dolma J, Jyot Singh J, Sehgal S (2021). Biodegradation of plastics: a state of the art review. Materials Today: Proceedings.

[ref-7] Bozek M, Hanus-Lorenz B, Rybak J (2017). The studies on waste biodegradation by *Tenebrio molitor*.

[ref-8] Brandon AM, Gao S-H, Tian R, Ning D, Yang S-S, Zhou J, Wu W-M, Criddle CS (2018). Biodegradation of polyethylene and plastic mixtures in mealworms (Larvae of *Tenebrio molitor*) and effects on the gut microbiome. Environmental Science & Technology.

[ref-9] Brandon AM, Garcia AM, Khlystov NA, Wu W-M, Criddle CS (2021). Enhanced bioavailability and microbial biodegradation of polystyrene in an enrichment derived from the gut microbiome of *Tenebrio molitor* (Mealworm Larvae). Environmental Science & Technology.

[ref-10] Bulak P, Proc K, Pytlak A, Puszka A, Gawdzik B, Bieganowski A (2021). Biodegradation of different types of plastics by *Tenebrio molitor* insect. Polymers.

[ref-11] Calmont B, Soldati F (2008). Biology and ecology of *Tenebrio opacus* Duftschmid, 1812; distribution and identification of French species belonging to the genus *Tenebrio* Linnaeus, 1758 (Coleoptera, Tenebrionidae). Revue de l’Association Roussillonnaise d’Entomologie.

[ref-12] Cao Q, Wickham JD, Chen L, Ahmad F, Lu M, Sun J (2018). Effect of oxygen on verbenone conversion from cis-verbenol by gut facultative anaerobes of *Dendroctonus valens*. Frontiers in Microbiology.

[ref-13] Cedervall T, Hansson L-A, Lard M, Frohm B, Linse S (2012). Food chain transport of nanoparticles affects behaviour and fat metabolism in fish. PLOS ONE.

[ref-14] Choe S, Kim Y, Won Y, Myung J (2021). Bridging three gaps in biodegradable plastics: misconceptions and truths about biodegradation. Frontiers in Chemistry.

[ref-15] Connat JL, Delbecque JP, Glitho I, Delachambre J (1991). The onset of metamorphosis in *Tenebrio molitor* larvae (Insecta, Coleoptera) under grouped, isolated and starved conditions. Journal of Insect Physiology.

[ref-16] Deia S (2019). The Story of Plastic (Documentary Film). San Francisco: The Story of Stuff Project. https://www.storyofstuff.org/movies/the-story-of-plastic-documentary-film/.

[ref-17] Dilkes-Hoffman L, Ashworth P, Laycock B, Pratt S, Lant P (2019). Public attitudes towards bioplastics –knowledge, perception and end-of-life management. Resources, Conservation and Recycling.

[ref-18] Engel P, Moran NA (2013). The gut microbiota of insects –diversity in structure and function. FEMS Microbiology Reviews.

[ref-19] Ganguly RK, Chakraborty SK (2024). Plastic waste management during and post Covid19 pandemic: challenges and strategies towards circular economy. Heliyon.

[ref-20] Goel V, Luthra P, Kapur GS, Ramakumar SSV (2021). Biodegradable/bio-plastics: myths and realities. Journal of Polymers and the Environment.

[ref-21] Gu CT, Li CY, Yang LJ, Huo GC (2014). Enterobacter *xiangfangensis* sp. nov., isolated from Chinese traditional sourdough, and reclassification of *Enterobacter sacchari* Zhu others,2013 as *Kosakonia sacchari* comb. nov. International Journal of Systematic and Evolutionary Microbiology.

[ref-22] He L, Yang S-S, Ding J, He Z-L, Pang J-W, Xing D-F, Zhao L, Zheng H-S, Ren N-Q, Wu W-M (2023). Responses of gut microbiomes to commercial polyester polymer biodegradation in *Tenebrio molitor* Larvae. Journal of Hazardous Materials.

[ref-23] Jiang S, Su T, Zhao J, Wang Z (2021). Biodegradation of polystyrene by *Tenebrio molitor*, Galleria *mellonella*, and *Zophobas atratus* larvae and comparison of their degradation effects. Polymers.

[ref-24] Jin L, Feng P, Cheng Z, Wang D (2023). Effect of biodegrading polyethylene, polystyrene, and polyvinyl chloride on the growth and development of yellow mealworm (*Tenebrio molitor*) larvae. Environmental Science and Pollution Research.

[ref-25] Kelaniyagama SH, Gannoruwa A, Nilmini A (2024). Synthesize and applications of biodegradable plastics as a solution for environmental pollution due to non-biodegradable plastics, a review. Sustainable Polymer & Energy.

[ref-26] Khairul Anuar SZ, Nordin AH, Nur Husna SM, Yusoff AH, Paiman SH, Md Noor SF, Nordin ML, Ali SN, Nazir Syah Ismail YM (2025). Recent advances in recycling and upcycling of hazardous plastic waste: a review. Journal of Environmental Management.

[ref-27] Kim MS, Chang H, Zheng L, Yan Q, Pfleger BF, Klier J, Nelson K, Majumder ELW, Huber GW (2023). A review of biodegradable plastics: chemistry, applications, properties, and future research needs. Chemical Reviews.

[ref-28] Krueger MC, Harms H, Schlosser D (2015). Prospects for microbiological solutions to environmental pollution with plastics. Applied Microbiology and Biotechnology.

[ref-29] Lakhiar IA, Yan H, Zhang J, Wang G, Deng S, Bao R, Zhang C, Syed TN, Wang B, Zhou R, Wang X (2024). Plastic pollution in agriculture as a threat to food security, the ecosystem, and the environment: an overview. Agronomy.

[ref-30] Liu J, Liu J, Xu B, Xu A, Cao S, Wei R, Zhou J, Jiang M, Dong W (2022). Biodegradation of polyether-polyurethane foam in yellow mealworms (*Tenebrio molitor*) and effects on the gut microbiome. Chemosphere.

[ref-31] Machona O, Chidzwondo F, Mangoyi R (2022). Tenebrio *molitor*: possible source of polystyrene-degrading bacteria. BMC Biotechnology.

[ref-32] Mini A, Prabhu VKK (1986). Significance of critical developmental stage on starvation induced endocrine mediated precocious metamorphosis in *Oryctes rhinoceros* (Coleoptera: Scarabaeidae). Proceedings: Animal Sciences.

[ref-33] Morales-Ramos JA, Rojas MG, Shapiro-Ilan DI, Tedders WL (2010). Developmental plasticity in *Tenebrio molitor* (Coleoptera: Tenebrionidae): analysis of instar variation in number and development time under different diets. Journal of Entomological Science.

[ref-34] Mukherjee S, Bhattacharyya A, Dar MA, Bisht R, Parvez A, Anani OA, Shahnawaz M, Dar MA, Daochen Z (2025). Challenges and perspectives for mitigating the footprints of plastic wastes produced by COVID-19 pandemic. Plastic and the COVID-19 pandemic: innovative solutions to mitigate plastic pollution.

[ref-35] Nayanathara T, Pilapitiya PGC, Ratnayake AS (2024). The world of plastic waste: a review. Cleaner Materials.

[ref-36] Newton J (1988). Insects and packaging —a review. International Biodeterioration.

[ref-37] Nikiema J, Asiedu Z (2022). A review of the cost and effectiveness of solutions to address plastic pollution. Environmental Science and Pollution Research.

[ref-38] Oliveira M, Ribeiro A, Hylland K, Guilhermino L (2013). Single and combined effects of microplastics and pyrene on juveniles (0+ group) of the common goby *Pomatoschistus microps* (Teleostei, Gobiidae). Ecological Indicators.

[ref-39] Ong SY, Zainab LI, Pyary S, Sudesh K (2018). A novel biological recovery approach for PHA employing selective digestion of bacterial biomass in animals. Applied Microbiology and Biotechnology.

[ref-40] Parashar N, Hait S (2021). Plastics in the time of COVID-19 pandemic: protector or polluter?. Science of The Total Environment.

[ref-41] Peng B-Y, Chen Z, Chen J, Yu H, Zhou X, Criddle CS, Wu W-M, Zhang Y (2020). Biodegradation of Polyvinyl Chloride (PVC) in *Tenebrio molitor* (Coleoptera: Tenebrionidae) larvae. Environment International.

[ref-42] Peng B-Y, Su Y, Chen Z, Chen J, Zhou X, Benbow ME, Criddle CS, Wu W-M, Zhang Y (2019). Biodegradation of polystyrene by dark (*Tenebrio obscurus*) and yellow (*Tenebriomolitor*) mealworms (Coleoptera: Tenebrionidae). Environmental Science & Technology.

[ref-43] Peng Y, Wu P, Schartup AT, Zhang Y (2021). Plastic waste release caused by COVID-19 and its fate in the global ocean. Proceedings of the National Academy of Sciences of the United States of America.

[ref-44] Pivato AF, Miranda GM, Prichula J, Lima JEA, Ligabue RA, Seixas A, Trentin DS (2022). Hydrocarbon-based plastics: progress and perspectives on consumption and biodegradation by insect larvae. Chemosphere.

[ref-45] Pradhan C, Satapathy K (2014). Decontamination of lead from aquatic environment by exploitation of floating macrophyte *Azolla microphylla* Kauf. IOSR Journal of Environmental Science, Toxicology and Food Technology (IOSR-JESTFT).

[ref-46] Prata JC, Silva ALP, Walker TR, Duarte AC, Rocha-Santos T (2020). COVID-19 pandemic repercussions on the use and management of plastics. Environmental Science & Technology.

[ref-47] Reddy RM (2008). Impact of soil composting using municipal solid waste on biodegradation of plastics. Indian Journal of Biotechnology.

[ref-48] Ren L, Men L, Zhang Z, Guan F, Tian J, Wang B, Wang J, Zhang Y, Zhang W (2019). Biodegradation of Polyethylene by *Enterobacter* sp. D1 from the Guts of Wax Moth *Galleria mellonella*. International Journal of Environmental Research and Public Health.

[ref-49] Restrepo-Flórez J-M, Bassi A, Thompson MR (2014). Microbial degradation and deterioration of polyethylene—a review. International Biodeterioration & Biodegradation.

[ref-50] Ross PA, Endersby NM, Hoffmann AA (2016). Costs of three *Wolbachia* infections on the survival of *Aedes aegypti* larvae under starvation conditions. PLOS Neglected Tropical Diseases.

[ref-51] Sanluis-Verdes A, Colomer-Vidal P, Rodriguez-Ventura F, Bello-Villarino M, Spínola-Amilibia M, Ruiz-Lopez E, Illanes-Vicioso R, Castroviejo P, Cigliano RAiese, Montoya M (2022). Wax worm saliva and the enzymes therein are the key to polyethylene degradation by *Galleria mellonella*. Nature Communications.

[ref-52] Sathiaseelan JJ, Afifah NMR, Abdullah AA-A, Ramakrishna S, Vigneswari S, Bhubalan K (2024). Exploring the advantages and limitations of degradation for various biodegradable micro-bioplastic in aquatic environments. Journal of Environmental Management.

[ref-53] Shafiei M, Moczek AP, Nijhout HF (2001). Food availability controls the onset of metamorphosis in the dung beetle *Onthophagus taurus* (Coleoptera: Scarabaeidae). Physiological Entomology.

[ref-54] Tao S, Li T, Li M, Yang S, Shen M, Liu H (2024). Research advances on the toxicity of biodegradable plastics derived micro/nanoplastics in the environment: a review. Science of The Total Environment.

[ref-55] Thayaparan M, Iqbal S, PK DC, Iqbal M (2013). Rhizofiltration of Pb by *Azolla pinnata*. International Journal of Environmental Sciences.

[ref-56] Tokiwa Y, Calabia BP, Ugwu CU, Aiba S (2009). Biodegradability of plastics. International Journal of Molecular Sciences.

[ref-57] Yang S-S, Ding M-Q, He L, Zhang C-H, Li Q-X, Xing D-F, Cao G-L, Zhao L, Ding J, Ren N-Q, Wu W-M (2021). Biodegradation of polypropylene by yellow mealworms (*Tenebrio molitor*) and superworms (*Zophobas atratus*) via gut-microbe-dependent depolymerization. Science of The Total Environment.

[ref-58] Yang S-S, Wu W-M, He D, Luo Y (2020). Biodegradation of plastics in *Tenebrio* Genus (Mealworms). Microplastics in terrestrial environments: emerging contaminants and major challenges.

[ref-59] Yang S-S, Wu W-M, Brandon AM, Fan H-Q, Receveur JP, Li Y, Wang Z-Y, Fan R, McClellan RL, Gao S-H, Ning D, Phillips DH, Peng B-Y, Wang H, Cai S-Y, Li P, Cai W-W, Ding L-Y, Yang J, Zheng M, Ren J, Zhang Y-L, Gao J, Xing D, Ren N-Q, Waymouth RM, Zhou J, Tao H-C, Picard CJ, Benbow ME, Criddle CS (2018). Ubiquity of polystyrene digestion and biodegradation within yellow mealworms, larvae of *Tenebriomolitor* Linnaeus (Coleoptera: Tenebrionidae). Chemosphere.

[ref-60] Yang B, Wu L, Feng W, Lin Q (2024). Global perspective of ecological risk of plastic pollution on soil microbial communities. Frontiers in Microbiology.

[ref-61] Yang X-G, Wen P-P, Yang Y-F, Jia P-P, Li W-G, Pei D-S (2023). Plastic biodegradation by in vitro environmental microorganisms and in vivo gut microorganisms of insects. Frontiers in Microbiology.

[ref-62] Yang J, Yang Y, Wu W-M, Zhao J, Jiang L (2014). Evidence of polyethylene biodegradation by bacterial strains from the guts of plastic-eating waxworms. Environmental Science & Technology.

[ref-63] Yang Y, Yang J, Wu W-M, Zhao J, Song Y, Gao L, Yang R, Jiang L (2015a). Biodegradation and mineralization of polystyrene by plastic-eating mealworms: part 1, chemical and physical characterization and isotopic tests. Environmental Science & Technology.

[ref-64] Yang Y, Yang J, Wu W-M, Zhao J, Song Y, Gao L, Yang R, Jiang L (2015b). Biodegradation and mineralization of polystyrene by plastic-eating mealworms: part 2, role of gut microorganisms. Environmental Science & Technology.

[ref-65] Yoshida S, Hiraga K, Takehana T, Taniguchi I, Yamaji H, Maeda Y, Toyohara K, Miyamoto K, Kimura Y, Oda K (2016). A bacterium that degrades and assimilates poly(ethylene terephthalate). Science.

[ref-66] Zhang Z, Peng H, Yang D, Zhang G, Zhang J, Ju F (2022). Polyvinyl chloride degradation by a bacterium isolated from the gut of insect larvae. Nature Communications.

[ref-67] Zhu J, Wang C (2020). Biodegradable plastics: green hope or greenwashing?. Marine Pollution Bulletin.

