# Supplementary figures and images for: Biodegradation of plastic waste by yellow mealworms (Tenebrio molitor larvae)
Source: PeerJ. 2026 Jan 8;14:e20429. doi: 10.7717/peerj.20429 (PMC12790784; doi:10.7717/peerj.20429)

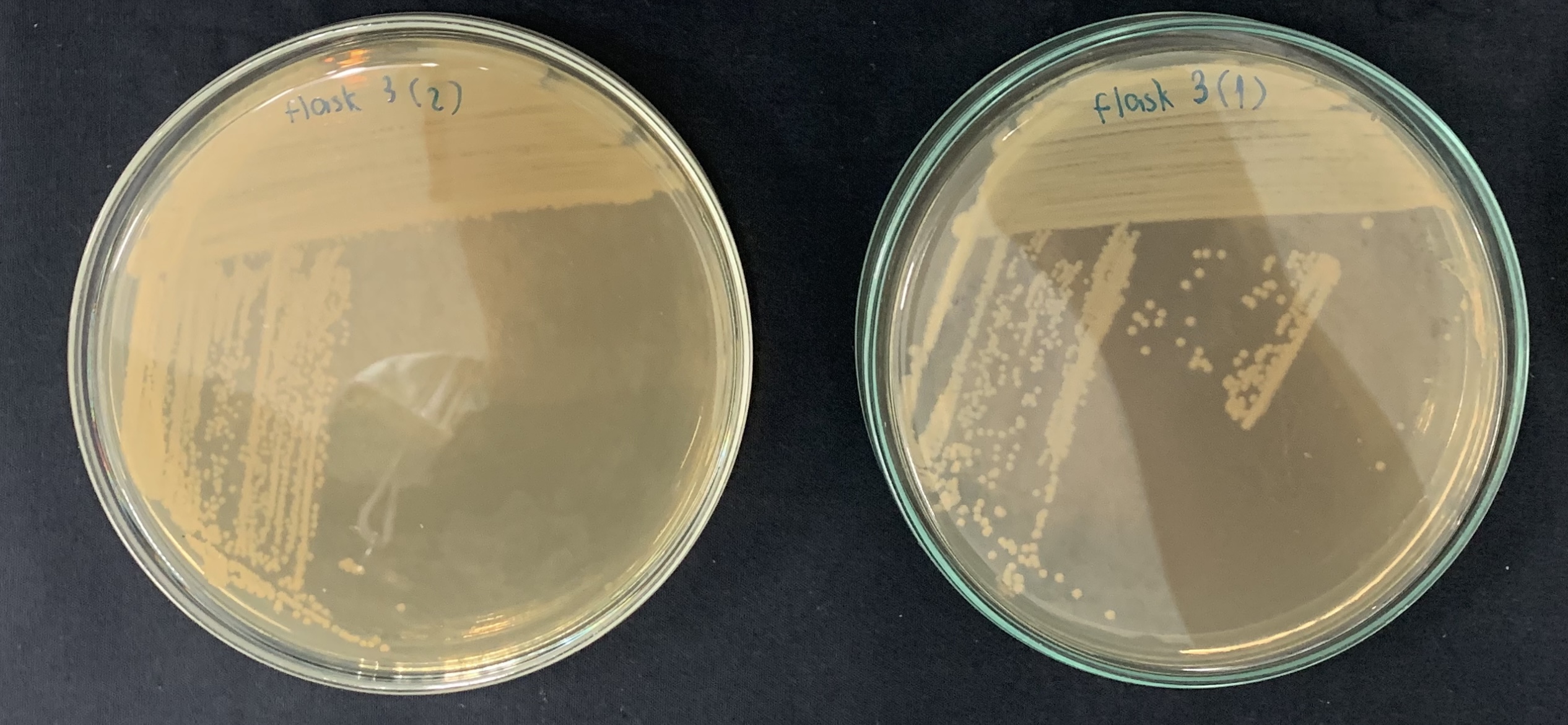

Supplement: Supplemental Information 1 — A robust growth of MG06 colonies on nutrient agar following subculture from a liquid carbon-free basal medium flasks containing PVC after 60 days of incubation, supporting its potential to utilize PVC as a carbon source. No colony formation was observed in the negative control without PVC (not shown), confirming that survival was dependent on the presence of PVC. [file peerj-14-20429-s001.jpg]
